# Cerebral Venous Thrombosis in Young Adults: Unknown Origins

**DOI:** 10.7759/cureus.41970

**Published:** 2023-07-16

**Authors:** Hovra Zahoor, Ameer Hamza, Eboselumen Aigbe, Nilmarie Guzman, Payam Nabizadeh-Eraghi

**Affiliations:** 1 Internal Medicine, HCA Florida Orange Park Hospital, Orange Park, USA; 2 Pulmonary and Critical Care Medicine, HCA Florida Orange Park Hospital, Orange Park, USA

**Keywords:** venous thromboembolsim, cerebral venous and dural sinus thrombosis, venous thrombosis, mechanical thromb, stroke, cerebral venous sinus thrombosis (cvst)

## Abstract

Cerebral venous thrombosis (CVT) is a relatively rare neurological disorder that may result in significant morbidity if not diagnosed and managed promptly. The clinical presentation of CVT is nonspecific and highly variable with acute, subacute, or chronic onset. It most often presents as a headache but may present with focal neurological symptoms, symptoms of intracranial hypertension, or encephalopathy. The predisposing factors for CVT are mainly acquired and genetic hypercoagulable conditions. However, the epidemiology, predisposing factors, and clinical presentation of CVT are not clearly established given the rare nature of the condition. We present a case series of three young patients who did not have any classic underlying etiology for CVT or any prior diagnosis of venous thrombosis. We want to report this case series to show that a high index of suspicion should be maintained regardless of the absence of risk factors.

## Introduction

Cerebral venous thrombosis (CVT) is defined as a thrombus of the cerebral veins and/or cavernous sinuses. The incidence is 0.22-1.32/100,000/year [1], accounting for 0.5% of all strokes [2]. Since CVT is a rare clinical entity, it can be a challenge to diagnose.

Studies have shown that the incidence of CVT among adults is probably higher than previously believed [1]. The incidence is particularly high in young adults, with median age being 34 years in females and 42 years in males [3]. The incidence has also noted to be increasing in the past decades because of the improvement in neuroradiological techniques [4]. It has also been noted to have a more variable presentation than previously realized. In this case series, we present three cases of young adults without classic risk factors for CVT that were admitted to the intensive care unit of a community hospital in 2022.

## Case presentation

Case 1

**Figure 1:**
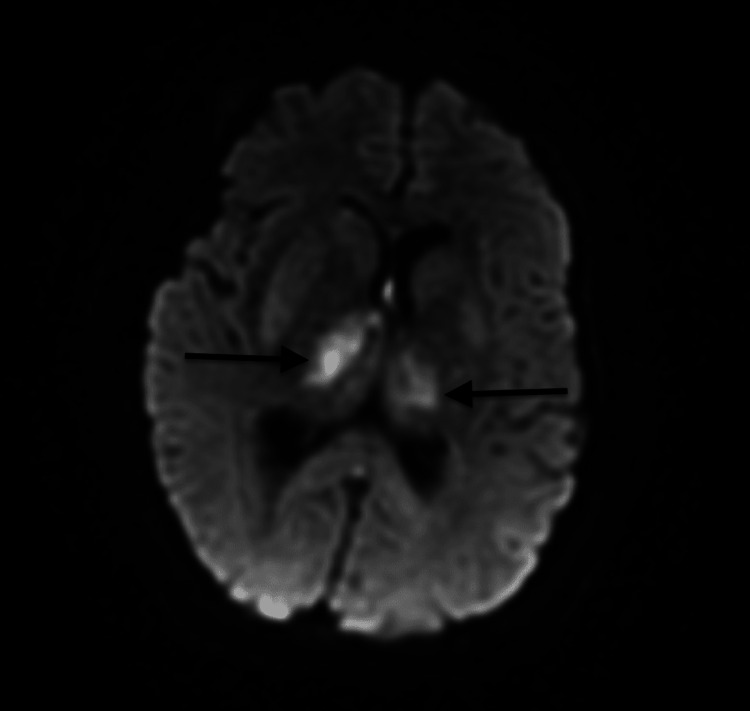
MRI brain (without contrast) MRI brain (without contrast) revealed secondary venous infarcts of bilateral thalami and right basal ganglia. The arrows show bilateral thalami and right basal ganglia venous infarcts.

A 25-year-old female with Smith Magenis Syndrome presented with complaints of lethargy, vomiting, diarrhea, and abdominal pain for a few days. She was diagnosed and managed for proctocolitis. However, on day two of admission patient was noted to be increasingly altered. A stroke alert was called. The neurologist recommended no tenecteplase as it was unclear when the patient was last seen at her baseline neurological status and whether or not she was within the window time period for tenecteplase. Computed Tomography (CT) brain without contrast revealed findings concerning CVT and venous infarct. A CT brain venogram revealed occlusion of the inferior sagittal sinus and bilateral deep cerebral veins. Magnetic Resonance Imaging (MRI) brain with and without contrast (Figure 1) revealed secondary venous infarcts of bilateral thalami and right basal ganglia in addition to CVT. The neuro-interventionalist recommended anticoagulation with intravenous (IV) unfractionated heparin infusion and no surgical intervention due to patency of the torcula and right transverse/sigmoid system. Hematology was consulted. Lupus anticoagulant; antiphospholipid, antinuclear, and antineutrophil cytoplasmic antibodies were negative. Fibrinogen and D-dimer were within normal limits. The patient’s Glasgow Coma Scale (GCS) worsened (8); she was intubated for airway protection. An electroencephalogram (EEG) revealed interictal discharges; therefore, levetiracetam was initiated. The patient had a gradual improvement in her neurological status and was extubated on hospital day 10. The patient was transitioned to apixaban prior to discharge to a rehabilitation facility. Extensive work-up as an outpatient was negative for any inherited or acquired hypercoagulable disorders. The patient was discharged from the rehabilitation facility with remarkable improvement.

Case 2

**Figure 2:**
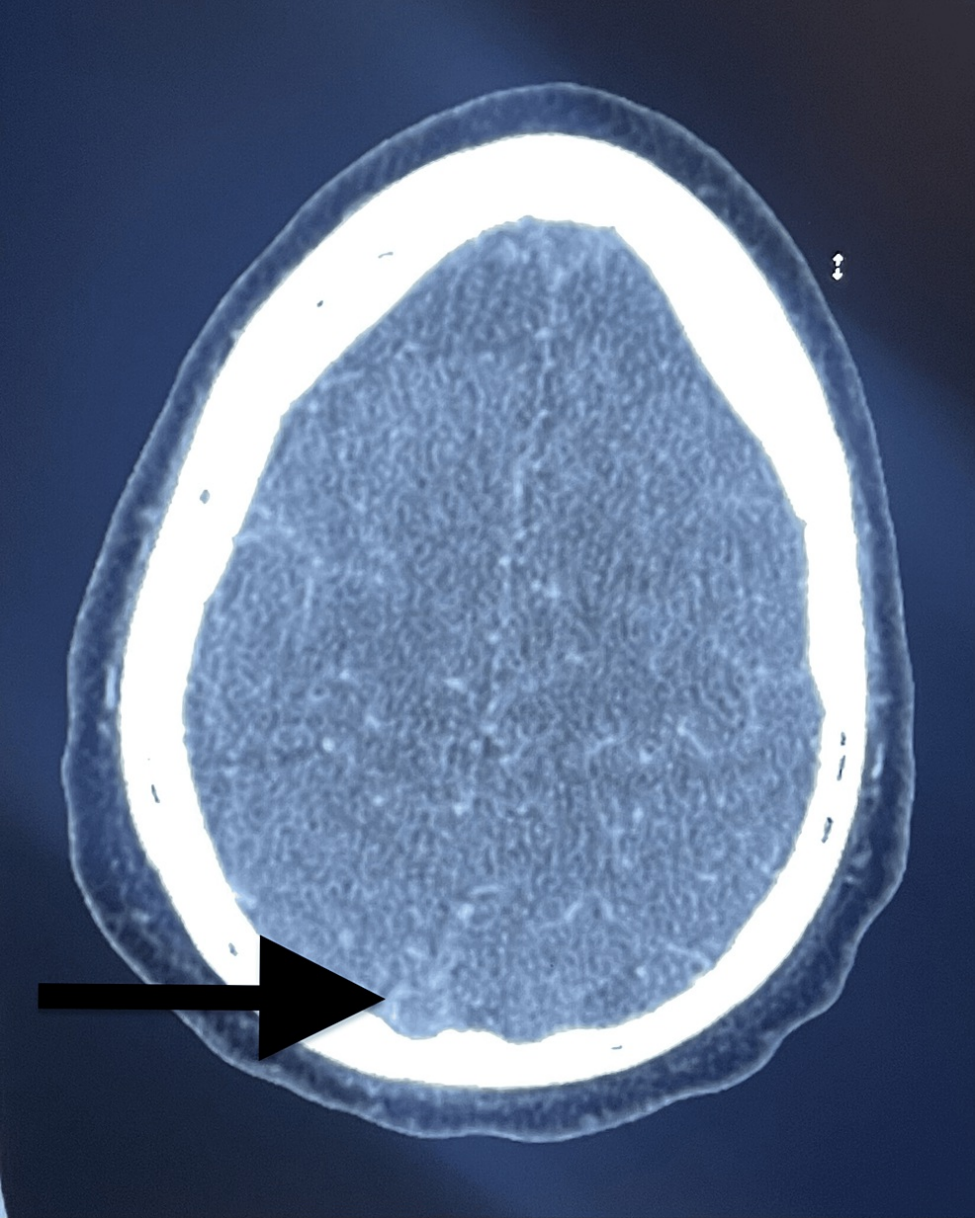
Head CT angiography image CT angiography of the head revealed a lack of enhancement at the confluence of sinuses (black arrow) concerning cerebral venous thrombosis.

A 45-year-old male with a past medical history of hypertension, headaches treated with gabapentin, obsessive-compulsive disorder, and major depressive disorder presented to the hospital via ambulance with a stroke code. The patient had a severe headache, right-sided hemiparesis, right-sided facial droop, and generalized numbness. A brain CT scan without contrast showed bilateral subarachnoid blood with no mass effect or midline shift. Head CT angiogram (Figure 2) was significant for findings concerning a venous sinus thrombosis with no aneurysm or vascular malformation. He was emergently taken to the catheterization laboratory where a superior sagittal sinus venogram demonstrated multiple filling defects and poor opacification of the right transverse sigmoid sinus and left transverse sigmoid sinus junction. A mechanical thrombectomy was performed. Post-intervention venogram and arteriogram showed improved patency of the right transverse sinus system and left transverse sigmoid sinus junction.

In the intensive care unit (ICU), the neurological examination showed that the patient was awake, alert, and oriented to self with slow verbal responses, intact cranial nerves, and mild generalized weakness without focal neurologic deficits. He was initiated on IV unfractionated heparin infusion. Post-thrombectomy brain CT without contrast demonstrated a slight interval increase in diffuse bilateral subarachnoid blood but no significant mass effect, hydrocephalus, or midline shift. Hematology was consulted. Hypercoagulability work-up with anti-cardiolipin, antinuclear and anti-phosphatidylserine antibodies as well as lupus anticoagulant profile were all unremarkable. Testosterone was within normal range. On hospital day 6, a brain MRI without contrast demonstrated unchanged multifocal subarachnoid hemorrhage and subacute infarct in the left thalamus and corona radiata. On hospital day 9, after a multidisciplinary discussion with hematology and neurology, he was transitioned to oral anticoagulation. Apixaban was initiated, 10 mg twice daily for 7 days followed by 5 mg twice daily indefinitely (given that the subarachnoid bleed remained stable). The patient remained neurologically stable through hospital day 19. He was discharged to inpatient short-term rehabilitation with instructions to follow up with hematology within 1-2 weeks of discharge as well as a subsequent head CT scan to monitor the subarachnoid hemorrhage.

Case 3

A 29-year-old female with a past medical history of migraines and anxiety disorder presented with complaints of headaches for the past three weeks and new onset generalized numbness. The patient had been seen in the Emergency Department (ED) on multiple occasions for headaches (without focal neurological symptoms) over the past several days. Further history revealed miscarriage (five weeks fetus) two and a half years ago, and she also reported being on estrogen-containing birth control pills.

**Figure 3:**
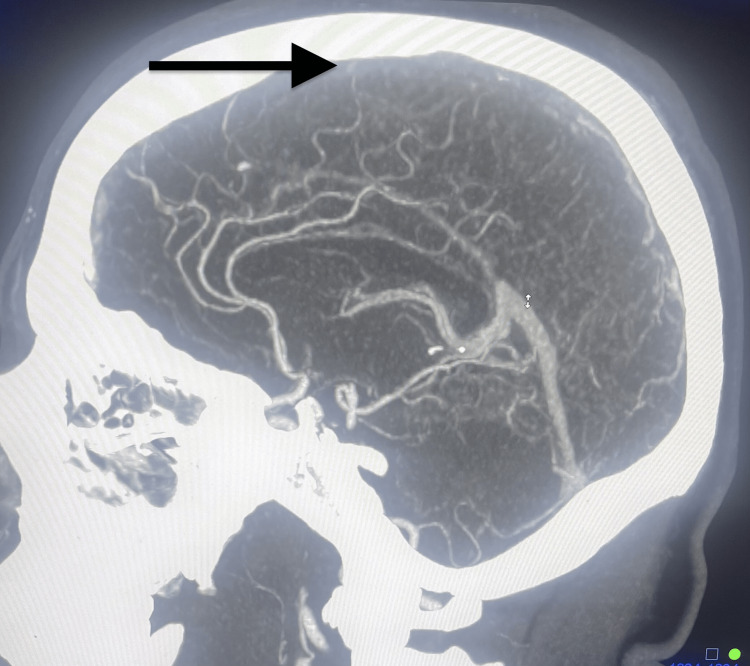
Head CT angiogram The angiogram shows a lack of enhancement in the superior sagittal sinus (arrows) concerning cerebral venous thrombosis.

In the ED, a few hours after the initial evaluation, the patient was noted to have word-finding difficulty but no focal neurological deficits. A stroke alert was called. The patient was outside of the tenecteplase window. A CT brain without contrast revealed extensive cerebral venous thrombosis. A CT angiogram head and neck (Figure 3) revealed extensive venous thrombus within the superior convexity cortical veins, superior sagittal sinus, left transverse sinus, left sigmoid sinus, and left jugular bulb. Normal arteries were noted. The interventional neurologist evaluated the patient, an IV unfractionated heparin infusion was started, and a thrombectomy was performed. The patient was admitted to the ICU post-thrombectomy as she required mechanical ventilation. IV heparin infusion was continued. The patient exhibited some seizure-like activity and was started on levetiracetam. EEG revealed no epileptiform features. MRI brain revealed venous infarcts of cerebral hemispheres, left more than right, without significant mass effect or hydrocephalus.

Hematology was consulted. The work-up revealed that factor VII, VIII, fibrinogen, and homocysteine were within normal range. Beta-2-GPI, antiphosphatidylserine and anticardiolipin antibodies were negative. However, given the severity of the event, lifelong anticoagulation was recommended.

The patient was successfully extubated on day 4 of admission. A repeat CT head showed no transformation to hemorrhage. The patient was thereby discharged to inpatient rehabilitation with apixaban 5 mg twice daily and further outpatient follow-up.

Table 1 illustrates hypercoagulable work-up performed inpatient for the three cases discussed above. All tests were collected prior to the start of therapeutic anticoagulation with continuous unfractionated heparin infusion or treatment doses of apixaban.

**Table 1:** Hypercoagulable work-up performed inpatient for the three cases discussed in this study All tests as above were collected prior to the start of therapeutic anticoagulation with continuous unfractionated heparin infusion or treatment doses of apixaban. Any values left blank means the test was not performed for that patient. PT: Prothrombin Time; INR: International Normalized Ratio; aPTT: Activated Partial Thromboplastin Time; TT: Thrombin Time; ACT: Activated Clotting Time; ANA: Antinuclear Antibodies

TESTS (normal range)	Case 1	Case 2	Case 3
PT (10.2 - 12.9 sec)	12.5 sec	12.5 sec	13.4 sec
INR	1.1	1.1	1.18
aPTT (25.1-36.5 sec)	33.2 sec	22.9 sec	29.1 sec
D-dimer (0 - 500 ng/mL)		3367 ng/mL	
TT (10.3 - 16.6 sec)	10.4 sec	16 sec	
ACT (112-144 sec)		165 sec	122 sec
Lupus anticoagulant PT (9.4 - 12.5 sec)		15.9 sec	
Lupus anticoagulant PTT (25.1 - 36.5 sec)		39.4 sec	
ANA		negative	
Fibrinogen			331 sec
Phosphatidylserine IgG (0-30 units)		9 units	
Phosphatidylserine IgA (0 - 19 units)		1 unit	
Phosphatidylserine IgM (0-30 units)		13 units	
anti-Cardiolipin IgG Ab (< 20 GPL U/mL)		< 1.6 GPL U/mL	
anti-Cardiolipin IgA Ab (< 20 GPL U/mL)		< 2.0 GPL U/mL	
anti-Cardiolipin IgM Ab (< 20 GPL U/mL)		< 1.5 GPL U/mL	
Factor VII			63%
Factor VIII			143%
Factor II G20210A variant			Not Detected
Factor X (77-131%)		84.40%	
Silica Clot Time Mix (0 - 1.16 Ratio)	0.96 Ratio		
Silica Clot Time Screen (0 - 1.16 Ratio)	1.2 Ratio		
Silica Clot Screen Ratio (0 - 1.6 Ratio)	0.93 Ratio		
Silica Clot Time Confirmation (0 - 1.6 Ratio)	1.29 Ratio		

## Discussion

We present three cases of young adults who were admitted to the intensive care unit of a community hospital in 2022 with cerebral venous sinus thrombosis (CVT) of unclear etiologies.

Given the recent pandemic, recent literature has focused on COVID-19 infection and/or its vaccine as possible etiologies for the development of CVT. Some have linked it to the development of thrombosis-thrombocytopenia syndrome following COVID-19 vaccination [5]. Thrombosis-thrombocytopenia syndrome and CVT were reported as very rare adverse events in patients who received SARS-CoV-2 adenoviral vector vaccines [6]. Further research did note that COVID-19 vaccines are safe for patients with a history of CVT [7].

Furthermore, studies prior to the pandemic noted that the incidence of CVT was more common than previously reported and recommended that future CVT incidence studies should include comprehensive capture and review of neuroimaging [8].

Several other studies also corroborate that the incidence of cerebral venous thrombosis among adults is higher than previously believed [1]. Careful consideration by radiologists regarding early signs of CVT on routine brain imaging is needed [2] as it will decrease the misdiagnosis of CVT. Some studies noted that the incidence of CVT is particularly high in young adults and that the incidence has increased in past decades because of the improvement of neuroradiological techniques [4]

This case series depicts a population of young adults (25 to 45 years of age) who developed CVT without clear etiologies or risk factors. Case 3 might represent an exception as she was a young female on oral contraceptive pills (OCP). However, a major thrombotic event in a young patient with otherwise no prior history of venous thromboembolism suggests the presence of an unidentified factor, even in the presence of years of OCP administration. Additionally, though all three patients were vaccinated for COVID-19, cases 1, 2, and 3 received different COVID-19 vaccinations, Johnson and Johnson, Pfizer, and Moderna, respectively. There was also significant variability regarding the timing of the vaccine, ranging from eight months to two years prior to presentation.

These three young patients with no known traditional risk factors ( acquired and genetic prothrombotic conditions, obesity, malignancy, central nervous system infections, head injury) and unclear CVT etiologies maintain that a high index of suspicion is needed when formulating differentials, especially in young adults, and be willing to obtain diagnostic imaging, such as computed tomography venograms and magnetic resonance venograms, in the workup of such patients.

In summary, data on CVT epidemiology is not well characterized. The quality of evidence is low and seems to stem from small, mostly single-center studies [9]. Further investigation is warranted- possibly in the form of large multi-centric, multinational databanks to increase our knowledge regarding the epidemiology of CVT. Through this, we also need to understand geographical, ethnic, socioeconomic, genetic, and environmental factors that may affect the incidence and severity of the disease. There are ongoing efforts to create such databases of CVT patients, as well as ongoing global studies that explore cerebral venous thrombosis genetics [9].

## Conclusions

Literature regarding the epidemiology of cerebral venous thrombosis is limited. This along with the variable clinical presentation of CVT poses a barrier to timely diagnosis. CVT is a life-threatening condition that if not diagnosed early on can lead to adverse outcomes. It is therefore imperative that more high-quality research is performed to optimize the available data regarding the epidemiology and clinical presentation of CVT. It is also important for physicians to hold a high index of suspicion for CVT in patients regardless of the presence of established risk factors.
